# ^113^Cd NMR Experiments Reveal an Unusual Metal Cluster in the Solution Structure of the Yeast Splicing Protein Bud31p**

**DOI:** 10.1002/anie.201412210

**Published:** 2015-02-20

**Authors:** Anne-Marie M van Roon, Ji-Chun Yang, Daniel Mathieu, Wolfgang Bermel, Kiyoshi Nagai, David Neuhaus

**Affiliations:** MRC Laboratory of Molecular BiologyFrancis Crick Avenue, Cambridge CB2 0QH (UK); Bruker BioSpin GmbHSilberstreifen, 76287 Rheinstetten (Germany)

**Keywords:** heteronuclear correlation, metal clusters, NMR spectroscopy, structure elucidation, zinc finger proteins

## Abstract

Establishing the binding topology of structural zinc ions in proteins is an essential part of their structure determination by NMR spectroscopy. Using ^113^Cd NMR experiments with ^113^Cd-substituted samples is a useful approach but has previously been limited mainly to very small protein domains. Here we used ^113^Cd NMR spectroscopy during structure determination of Bud31p, a 157-residue yeast protein containing an unusual Zn_3_Cys_9_ cluster, demonstrating that recent hardware developments make this approach feasible for significantly larger systems.

Tetrahedral co-ordination of zinc by cysteine and sometimes histidine residues is an extremely efficient way to impart additional rigidity to small protein structural domains. It parallels for intracellular proteins the role played by disulfide bridges in extracellular proteins, and very many zinc-binding domains with widely varying co-ordination patterns have been reported since the original proposal of the zinc finger motif.[1a,1b] When determining the solution structure of any protein where more than one structural zinc is bound, it is essential to establish which ligand binds to which zinc, but this can be difficult since zinc has no slowly relaxing (spin 1/2) NMR-active isotope, and nuclear Overhauser effect (NOE) connectivities and J-couplings involving other nuclei (^1^H, ^13^C, ^15^N) often provide little or no directly relevant data. For several small domains, it was early shown that substitution of zinc by a spin 1/2 isotope of cadmium (usually ^113^Cd) could largely overcome such problems, as [^1^H, ^113^Cd] correlation experiments could be used to identify directly the metal-binding topology. Most examples have involved metallothioneins, for example Refs. [2] and [3], but others have included Gal4,[4a–c] Lac9,[5] rubredoxin,[6] glucocorticoid receptor DBD,[7] HIV p7,[8] and two RING finger proteins.[9a,9b] In all such cases of which we are aware, the size of the protein domain under study has been less than 100 residues, and in the great majority less than 50; for instance, the two independently folded domains present in most metallothioneins studied by ^113^Cd NMR spectroscopy each comprise approximately 30 residues. Although we know of no systematic study of factors affecting sensitivity of ^113^Cd correlation experiments, and while the relationship between molecular weight and observed linewidth may sometimes be complicated by exchange effects,[10a,10b] these examples strongly imply that poor sensitivity makes ^113^Cd correlation experiments challenging for protein domains larger than about 10 kDa, which may be why this approach has been surprisingly little used. In this work we have exploited recent improvements in spectrometer hardware, most particularly introduction of cryogenically cooled probeheads capable of measuring ^113^Cd correlation spectra with much enhanced sensitivity, to help solve the solution structure of a 157-residue, 18.5 kDa yeast protein, Bud31p, that contains a highly unusual 3-metal cluster, thereby demonstrating that these technical improvements now make it feasible to use the cadmium substitution approach for larger systems.

Bud31p is highly conserved in all eukaryotes (43 % identity from yeast to human) and is thought to be important in promoting efficient pre-mRNA splicing.[11] Pull-down studies have shown that it is part of the NTC-related complex.[12a,b] Though it is not essential, null mutants in *S. cerevisiae* show severe abnormalities in cytoskeleton, actin distribution, and Bud formation,[13] while knockdown of Bud31 in cell-based RNAi screens in fly and human cell cultures also caused severe effects.[14] Mass spectrometry clearly showed that native Bud31p contains three zincs (see Figure S1 in the Supporting Information), while analytical gelfiltration (Figure S2) and ultracentrifugation (data not shown) showed it to be a monomer in solution. Sequence analysis (Figure S3) revealed nine absolutely conserved cysteines that might bind zinc, although no homology to any known zinc finger motif was identified; in addition, the *S. cerevisiae* sequence contains four histidines with varying degrees of conservation, one or more of which might also bind zinc.

In a first approach to determining metal-binding topology, we carried out a conventional NMR structure determination of Bud31p, using protein recombinantly expressed in *E. coli* and without specifying any bound metal ions or ligand–ligand interactions. An essentially full resonance assignment was achieved,[15] and structural constraints based on NOE contacts and J-couplings derived, including specification of the χ^1^ rotamer for 57 residues including eight of the nine cysteine residues and three of the four histidine residues. Preliminary structure calculations (data not shown) revealed the overall fold, which comprises an N-terminal four-helix bundle rigidly joined to an irregular C-terminal domain containing all of the conserved cysteines and the three most highly conserved histidines. However, these data did not establish the metal-binding topology of Bud31p, or even whether the histidines participate. Without such knowledge, determination of an atomic resolution solution structure is impossible, so we turned to ^113^Cd NMR experiments. ^113^Cd_3_ Bud31p was prepared by slow demetallation of native Bud31p with excess EDTA followed by equilibration with ^113^Cd on ice; mass spectrometry confirmed complete substitution, far-UV CD spectra showed the overall structure is retained, and 1D ^113^Cd NMR spectra showed three resonances (Figures S1, S4, and S5).

A [^113^Cd-^113^Cd] COSY experiment acquired for 2.5 days using the cryogenically cooled probehead showed cross-peaks linking all three cadmium signals, albeit the different linewidths of the cadmium signals resulted in substantially different intensities amongst the cross-peaks because of anti-phase cancellation (Figure 1 a). These key connectivities establish unambiguously that the three metals present in Bud31p form a cyclic cluster, and are consistent with 2-bond coupling pathways of the type ^113^Cd-S(Cys)-^113^Cd. This in turn implies that three of the cysteine residues each bridge between two metals, and shows that the nine absolutely conserved cysteine residues of Bud31p are alone sufficient to complete the tetrahedral co-ordination of three metals without any need to invoke involvement of further ligands such as histidine (Figure 2 a).

**Figure 1:**
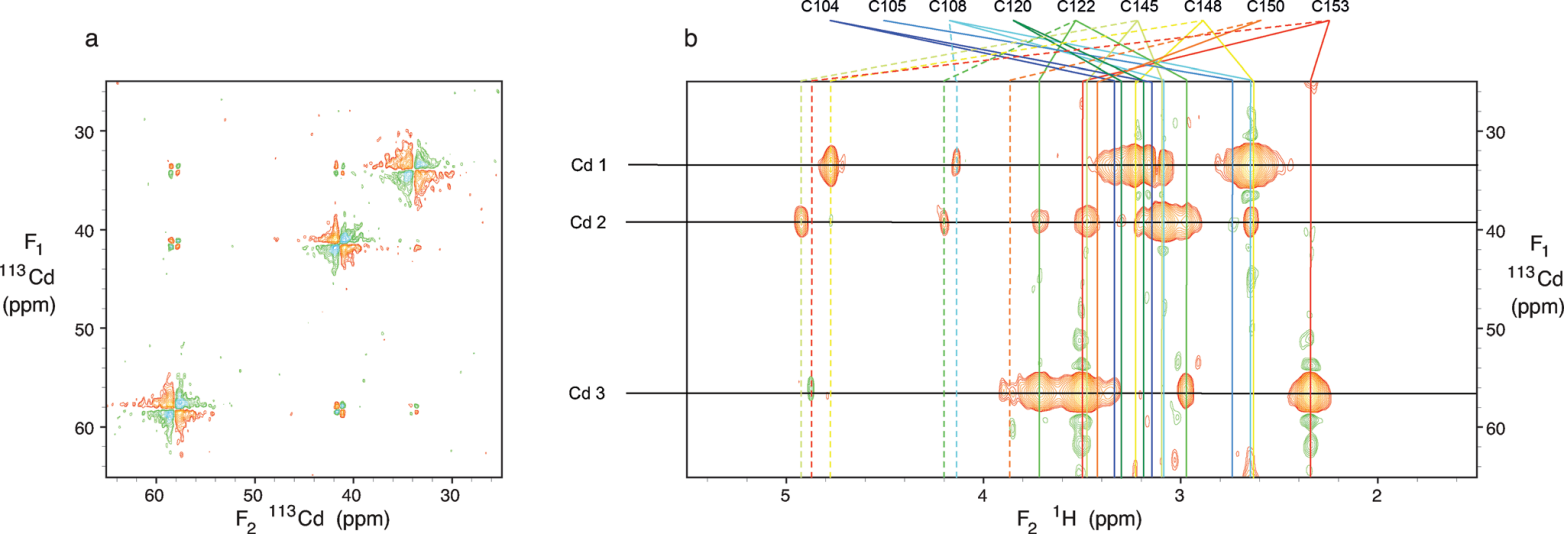
a) [ ^113^Cd-^113^Cd] COSY and b) [ ^113^Cd-^1^H] HMQC-RELAY spectra of ^113^Cd_3_ Bud31p (2.6 mm in D_2_O). Assignments of Cys Hβ (solid lines) and Hα (dotted lines) signals are shown in (b).

**Figure 2:**
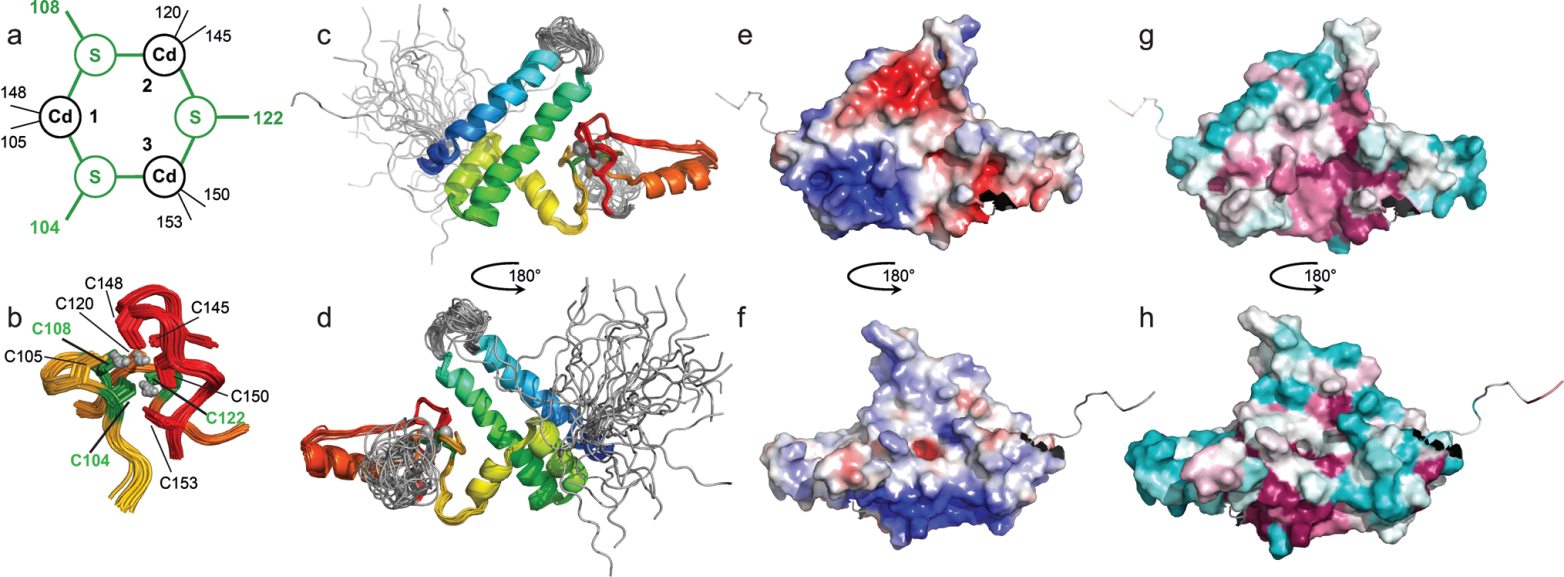
Solution structure of Bud31p. a) Metal-binding topology as established by ^113^Cd NMR experiments; bridging cysteines are shown in green (see also Figure S7). b–d) Final structural ensemble, showing b) metal cluster (parts of backbone omitted for clarity) and c,d) full structure (chainbow coloring, disordered regions in gray). e–h) Lowest NOE energy structure, showing e,f) electrostatic surface and g,h) conservation surface (cyan, variable; maroon, conserved); see also Figure S8 for hydrophobic surface and S3 for conservation scores displayed on the sequence. In views (e–h) the disordered tails are omitted from the calculated surfaces for clarity.

In order to assign individual connectivities between cysteines and metals, we used [^1^H, ^113^Cd] correlation experiments. Since cadmium substitution causes chemical shift perturbations for nuclei near the metal cluster, a partial reassignment was carried out using a combination of 2D NOESY, 2D TOCSY and natural abundance [^13^C, ^1^H] HSQC data. Interpretation of simple [^113^Cd, ^1^H] HMQC experiments was severely hampered by overlap amongst the cysteinyl Hβ protons, making it necessary to employ additional experiments to transfer magnetization from these protons to others. To achieve this we used HMQC-RELAY experiments[3] (e.g. Figure 1 b; 22 hour experimental time), which transfer magnetization through Hα–Hβ and Hβ–Hβ J-couplings, and HMQC-NOESY experiments[16] (e.g. Figure S6; 31 hour experimental time). Cross-peaks to cysteinyl Hα signals were very useful, as these have narrower multiplet structures and are better dispersed than the Hβ signals; additional cross peaks to Hβ protons, presumably missing from simple HMQC spectra due to small values of *J*(^113^Cd, ^1^Hβ), were also important. Data acquisition at two temperatures (25 °C and 35 °C) helped resolve ambiguities and improve sensitivity, although this approach was limited by reduced protein stability at the higher temperature (Figure S4). Combined interpretation of all data gave the metal-binding topology shown in Figure 2 a and Figure S7, which was used to define covalent bonding constraints during a final run of structure calculations. This gave the structure shown in Figure 2 b–h and Figure S8, with statistics in Table S1 and Figure S9.[17]

The metal cluster is superficially similar to three-metal clusters in metallothioneins and SET domains, in that all involve a six-membered metal–sulfur ring formed by three metal and three sulfur atoms, but the sequential identity of the bridging cysteines and overall binding topology in Bud31p are quite different, and as far as we are aware represent a novel arrangement. The calculated structures all show the metal–sulfur ring in a chair conformation, though given that this is determined only indirectly by the NMR measurements and force field, it should not be taken as definitive. Disordered parts of the structure correspond to regions of genuine flexibility; as well as the N-terminal tail, the two disordered loops (residues 37–44 and 108–120) correspond to very clearly distinguished regions of low ^15^N{^1^H} NOE values (Figure S10). The projecting helix (residues 125–135) and part of the following extended strand also show slightly reduced NOE values, suggesting a degree of relative motion in this part of the structure.

It is very likely that Bud31p functions as part of a larger complex, suggesting that some or all of the disordered regions may become stably folded in the context of forming interaction interfaces with as yet unidentified binding partners. Examination of the sequence conservation data (Figure S3) shows that the strongest conservation is for residues directly involved in maintaining the structure, including the metal-binding cysteines and residues involved in the predominantly hydrophobic contacts between structural elements. However, examination of the potential surface (Figure 2 e and f) reveals a strongly basic patch contributed mainly by residues Lys89, Lys94, Arg96, Lys97, and Arg123 that is highly conserved, suggesting that it could have functional significance. Acidic and basic patches on the opposite face of the protein (residues Asp25, Asp32, Asp36, Glu47 and Glu51 and residues Arg59, Arg61, Lys69, Arg70, Lys71 and Lys75, respectively) are somewhat less conserved, and additionally there is a hydrophobic patch involving mainly residues Ile121, Val124, Pro125, Leu129, Val140, Phe142 and Val146, most of which are strongly conserved; these could also be interaction patches. Intriguingly, examination of alignment data for the cysteine-rich metal-binding domain alone reveals that in a subset of species, mainly protozoa, sequences with a corresponding pattern of cysteines are found as tandem repeats, and lacking the helical domain (Figure S11). Although the structural significance of this finding remains unclear, it supports our interpretation that the structure of Bud31p is bipartite and that the novel metal cluster domain may occur independently of the helical bundle domain in other proteins. Very recently, it was reported that a short peptide from human Bud31p can act as a co-regulator of androgen receptor (AR).[18] Crystal structures (e.g. 4ODE) of AR in complex with this and related peptides showed the bound peptides form characteristic interactions from an FxxLF motif (or in the case of Bud31p, an FxxFY motif) in a helical conformation. However, while the corresponding residues of our structure (64–68) are also helical, superposition with 4ODE shows that full-length Bud31p could not bind to AR in the same way as the peptide unless it undergoes very large conformational changes. It remains to be seen how these observations can be reconciled.

In conclusion, this work has demonstrated that the determination of metal-binding topology using cadmium correlation experiments can now be applied for proteins significantly larger than those reported hitherto, which should allow for more reliable structure determination in such cases.

In memory of Detlef Moskau
