# Supplementary material for: 113Cd NMR Experiments Reveal an Unusual Metal Cluster in the Solution Structure of the Yeast Splicing Protein Bud31p
Source: Angew Chem Int Ed Engl. 2015 Feb 20;54(16):4861–4. doi: 10.1002/anie.201412210 (PMC4471582; doi:10.1002/anie.201412210)
Supplement: Supplementary file 1 [file anie0054-4861-sd1.pdf]

Angewandte Chemie
Eine Zeitschrift der Gesellschaft Deutscher Chemiker

Supporting Information

^{113}Cd NMR Experiments Reveal an Unusual Metal Cluster in the Solution Structure of the Yeast Splicing Protein Bud31p**

*Anne-Marie M. van Roon, Ji-Chun Yang, Daniel Mathieu, Wolfgang Bermel, Kiyoshi Nagai, and David Neuhaus**

anie_201412210_sm_miscellaneous_information.pdf

Supporting Information

SI.1) Materials and Methods

SI.1.1 Cloning, expression and purification of Bud31p.

The Bud31 coding gene was amplified by PCR using yeast genomic DNA. The Bud31 gene was cloned using BamHI and HindIII restriction sites into a pRK172 vector, in which hexa-histidine tagged glutathione-S-transferase and a tobacco etch virus protease cleavage site had been cloned between NdeI and BamHI restriction sites immediately upstream of the protein coding region. The pRK172-GST-Bud31 plasmid was transformed into BL21(DE3)RIL CodonPlus cells (Stratagene). The protein was either expressed in rich medium or in M9 minimal medium supplemented with $^{15}NH_4Cl$ or $^{15}NH_4Cl$ and [^{13}C]-glucose to obtain ^{15}N or ^{15}N-^{13}C isotope labelled protein, respectively, for NMR experiments. The GST-Bud31p fusion protein was expressed overnight at 37°C after induction with 0.5mM IPTG. Upon induction the medium was supplemented with 0.1 mM $ZnCl_2$. Harvested cells were resuspended in glutathione-binding buffer (20 mM Tris-HCl pH 7.4, 500 mM Urea, 500 mM NaCl, 1 μM $ZnSO_4$ and 5 mM β-mercaptoethanol), containing complete protease inhibitor cocktail (Roche) and lysed by sonication. The lysate was cleared by centrifugation, the supernatant loaded on glutathione-Sepharose (GE-Healthcare) equilibrated with glutathione-binding buffer and the protein was eluted by a 0-20 mM linear gradient of glutathione. Peak fractions were incubated overnight with His-tagged tobacco etch virus protease at room temperature while dialysing against Ni-NTA binding buffer (20 mM Tris-HCl pH 7.4, 500 mM Urea, 500 mM NaCl, 25 mM imidazole, 1 μM $ZnSO_4$ and 5 mM β-mercaptoethanol). After complete cleavage the sample was loaded on Ni-NTA agarose (Qiagen) to remove His-tagged tobacco etch virus protease, His-tagged glutathione-S-transferase and minor protein contaminants. The flowthrough containing Bud31p was loaded immediately onto Hydroxyapatite (HA) (Bio-Rad)

and eluted with a 0-6% linear gradient of ammonium sulfate in HA buffer (20 mM Na-phosphate buffer pH 7.2, 200 mM NaCl, 5 mM β-mercaptoethanol), peak fractions were pooled and Bud31p was concentrated and buffer exchanged using an Amicon-Ultra-15 concentrator (Millipore) with an exclusion size of 10 kDa.

SI.1.2 Biophysical characterisation of Bud31p.

Analytical gelfiltration was performed on a Superdex S200 HR 10/300 column with 20 mM Tris-HCl pH 7.4, 200 mM NaCl and 1 mM DTT as eluent. Analyical ultracentrifugation was carried out using a Beckman XL-A centrifuge equipped with an absorbance detection system. Measurements were carried out at 30 and 40 krpm.

Mass spectrometry measurements were carried out on a Micromass LCT Mass Spectrometer, a time-of-flight (TOF) mass spectrometer equipped with both electrospray (ESI) and atmospheric pressure chemical ionization (APCI) ion source (Waters). The mass of Bud31p was measured either under denaturing conditions using 1% formic acid in 50% methanol or under native conditions using 150 mM ammonium acetate with 1 mM DTT.

Far-UV CD. Spectra of 0.2 mg/mL Zn_3 Bud31p or $^{113}Cd_3$ Bud31 in CD buffer (10 mM Na-phosphate pH 7, 50 mM KF) were recorded using a Jasco J-810 spectropolarimeter (Jasco Corp.) at 20°C in the spectral range from 190 to 260 nm with 0.2 nm resolution. Spectra were recorded as averages of six scans at a scanning speed of 100 nm/min. The response time was 2.0 s with a bandwidth of 1.0 nm. Quartz cuvettes with an optical path of 1 mm were used. The spectra were corrected for the buffer background.

SI.1.3 NMR experiments with Zn_3 Bud31p.

NMR samples of Zn_3 Bud31p comprised 0.3-0.5 mM (^{15}N-labelled) or 0.3-0.5 mM (^{15}N, ^{13}C-labelled) solutions (with additional vector-derived residues Gly-Gly-Ser- at the N-terminus in place of the native residue Met 1) in NMR buffer (20 mM Na-phosphate pH 6.5, 1 mM [$^2H_6$] DTT, 150 mM NaCl) in either H_2O (or ^2H_2O where stated below). All NMR data for Zn_3 Bud31p were acquired using Bruker Avance 800, DMX600 and DRX500 spectrometers (Bruker BioSpin GmbH), each equipped with a cryogenically cooled triple resonance ($^1H/^{15}N/^{13}C$) 5mm probe. Most experiments were conducted at 27°C and ^1H chemical shifts were calibrated using sodium 3,3,3-trimethylsilylpropionate (TSP) as an external ^1H reference; ^{15}N and ^{13}C chemical shifts were indirectly referenced to the ^1H shifts using the ratio of gyromagnetic ratios.[1] The following datasets were acquired: 2D: [^{15}N-^1H] HSQC, [^{13}C-^1H] HSQC covering the full ^{13}C spectral width, constant-time [^{13}C-^1H] HSQC covering only the aliphatic ^{13}C region, constant-time [^{13}C-^1H] HSQC covering only the aromatic ^{13}C region, [^1H-^1H] NOESY experiments (τ_m = 150 ms); 3D: HNCACB, HNCOCACB, HBHANH, HBHACONH, [^1H-^{13}C-^1H] HCCH-COSY, [^1H-^{13}C-^1H] HCCH-TOCSY, [^{13}C-^{13}C-^1H] HCCH-TOCSY, HNHB, HACAHB (^2H_2O), ^{15}N NOESY-HSQC (τ_m = 50 ms and 150 ms), ^{13}C NOESY-HSQC (τ_m = 50 ms and 150 ms; separate datasets acquired for ^{13}C aliphatic and aromatic spectral regions). Based on these experiments, a complete backbone assignment and a near complete side-chain assignment was obtained.

SI.1.4 NMR experiments with $^{113}Cd_3$ Bud31p.

To prepare $^{113}Cd_3$ Bud31p, essentially complete substitution of zinc by ^{113}Cd was achieved by slow de-metallation of native Bud31p with a 10-fold excess of EDTA at 0°C for 2 days followed by 10-fold dilution into a buffer (20 mM Tris pH 7.4, 200 mM NaCl, 50 mM

β-mercaptoethanol) containing a 25-fold excess of $^{113}CdCl_2$ and subsequent equilibration for 1 day at 0°C. Excess $^{113}CdCl_2$ was removed by buffer exchange to 20 mM Tris pH 7.4, 200 mM NaCl. For NMR experiments, $^{113}Cd_3$ Bud31p (natural abundance of ^{14}N and ^{12}C) was exchanged into NMR buffer with 5mM [$^2H_6$] DTT in ^2H_2O and concentrated using an Amicon-Ultra-15 concentrator (Millipore) with an exclusion size of 10 kDa, resulting in a final sample concentration of 2.6 mM.

NMR experiments were run as indicated below on either a Bruker AVIII HD 500 MHz spectrometer equipped with a cryogenically cooled 5mm probe having an inner broadband coil tuned to ^{113}Cd and outer ^1H coil, or a Bruker AVIII 850 MHz MHz spectrometer equipped with a cryogenically cooled triple resonance (^1H/^{15}N/^{13}C) 5mm probe, or a Bruker AVI 800 MHz spectrometer equipped with a cryogenically cooled triple resonance (^1H/^{15}N/^{13}C) 5mm probe: Experiments were conducted at either 25°C or 35°C, and ^{113}Cd chemical shifts were indirectly referenced to the ^1H shifts using the ratio of gyromagnetic ratios (in the case of cadmium chemical shifts, this process used the Bruker-defined absolute frequency scale for ^{113}Cd, based on $CdMe_2$ as a standard as listed in reference[2]). The following datasets were acquired at 25°C (and also at 35°C where indicated below) [^{113}Cd, ^{113}Cd] experiments, (run on AVIII HD 500): 2D phase-sensitive proton-decoupled COSY (1024 scans, 59 hour experiment time; see also below); [^{113}Cd, ^1H] experiments (run on AVIII 500): 2D HMQC, 2D HSQC, 2D HMQC-RELAY (also at 35°C; 2048 scans, 22 hour experiment time; see also below),[3] 2D HMQC-NOESY (2048 scans, 31 hour experiment time; see also below),[4] [^1H, ^1H] experiments, (run on AVIII 850 or AVI 800): 2D TOCSY (τ_m = 20 ms), 2D NOESY (τ_m = 100 ms); [^{13}C, ^1H] (natural abundance) experiments (run on AVIII 850 MHz): 2D HSQC (also at 35°C).

The [^{113}Cd, ^{113}Cd] phase-sensitive COSY was acquired with 64 complex points in t_1 (t_{1max} = 9.51 ms) and 4K complex points in t_2 (t_{2max} = 614.4 ms), of which only the first 256

were used during processing (t_{2max}(effective) = 38.4 ms). The recycle delay for inter-scan relaxation was 1.0s, a sine-bell-squared window function shifted by $\pi/3$ was applied in both time dimensions prior to Fourier transformation, and the frequency domain data size was 1K in F_1 and 4K in F_2. These values were not systematically optimized, but probably represent a reasonable compromise; the ^{113}Cd T_2 was probably in the region of 2–3 ms based on the observed 1D ^{113}Cd linewidth of approx. 100–150 Hz, and typical values of 2-bond (^{113}Cd, ^{113}Cd) J-couplings through cysteinyl sulfur are in the range 30–50 Hz,[5] which would imply a maximum in the transfer function $\sin(\pi J t_1).\exp(-t_1/T_2)$ also at approximately 2–3 ms. It is quite likely that ^{113}Cd transverse relaxation is dominated by the CSA mechanism (even though to a first approximation each cadmium ion experiences a symmetrical, tetrahedral bonding environment), suggesting that lower B_0 fields may offer some advantage for these experiments. However, at the time of writing the cryogenically cooled probe that we used for this work is only available for 500 MHz spectrometers, so the actual field-dependence of sensitivity could not be investigated. Broadening of the ^{113}Cd lines by conformational exchange might also contribute to the ^{113}Cd linewidth.

Optimization of the ^{1}H-detected [^{113}Cd, ^{1}H] correlation experiments is more complicated than for the COSY experiment, as it depends mainly on the values of the different 3-bond (^{113}Cd, ^{1}H) couplings that participate in the magnetization transfer pathways. These values are not known *a priori*, but it is very likely that they span a considerable range as they follow a Karplus-type dependence on cysteinyl $\chi2$ angle, with values of 0–75 Hz having been reported.[6] Given this expected complexity, we took an entirely empirical approach to optimization, carrying out short trial experiments using different values of the fixed transfer delays and also comparing HMQC-based and HSQC-based pulse sequences. We found that for HMQC experiments, transfer delays of 6.66 ms on each side of t_1 gave the best sensitivity of those values tested (3.33, 6.66, 5, 10 and 20 ms). HSQC gave markedly lower sensitivity; when using a transfer delay of 6.66 ms,

corresponding peaks in the HSQC spectrum were between roughly 40% and 70% less intense than in HMQC. It was possible to increase the sensitivity of the HSQC experiments for the most intense peaks by shortening the transfer delays, e.g. to 3.33ms, but as would be expected this significantly reduced the number of observable correlations since it discriminates against correlations through smaller (^{113}Cd, ^{1}H) couplings. No HSQC-based experiments were used in the final data used for structure determination. For the HMQC-RELAY experiment, the transfer delay has to accommodate evolution not only of (^{113}Cd, ^{1}Hβ) couplings but also of (^{1}Hα, ^{1}Hβ) couplings, the latter being significantly smaller than the former; in this case we found that, for relayed peaks observed on Hα signals, transfer delays of 10 ms on each side of t_1 gave the best sensitivity of those values tested (6.66, 10 and 15 ms). We also found that setting a higher sample temperature (308K) gave significantly improved sensitivity, presumably due to ^{113}Cd line-narrowing. For the HMQC-NOESY experiment we employed the same transfer delays (6.66 ms) as in the simple HMQC case, and an NOE mixing time of 75 ms. Data for [^{113}Cd, ^{1}H] correlation experiments were acquired with 16 complex points in t_1 (t_{1max} = 3.16) and 1K complex points in t_2 (t_{2max} = 127.8). The recycle delay for inter-scan relaxation was 1.0s (HMQC-RELAY) or 1.5s (HMQC-NOESY), only the first 512 complex points in F_2 were used during processing (t_{2max}(effective) = 63.9 ms), a sine-bell window function shifted by $\pi/3$ was applied in both time dimensions prior to Fourier transformation, and the frequency domain data size was 1K in F_1 and 4K in F_2.

Although practical considerations such as sample lifetime limited our ability to quantitate relative sensitivities between different spectrometers, early ^{113}Cd NMR experiments carried out with similar samples using a conventional (room temperature) inverse-geometry broadband probe at 600 MHz and 27°C showed that only very few connectivities (and none involving relayed ^{113}Cd-^{1}H-^{1}H transfers) could be detected even with very long experiment times, and these were completely insufficient to establish the

metal binding topology; this shows the practical necessity of using the newer hardware when attempting to solve this structure.

SI.1.5 Determining the metal-binding topology for $^{113}Cd_3$Bud31p.

Resonance assignments of Cysteine residues in $^{113}Cd_3$ Bud31p were based on analogy with the assigned signals from Zn_3 Bud31p, by analyzing patterns of cross-peaks in [^{13}C-^{1}H] HSQC, [^{1}H-^{1}H] TOCSY (τ_m = 20 ms) and NOESY (τ_m = 100 ms). The cysteine CβH_2 groups of the Zn-bound protein all gave well-resolved pairs of signals in the [^{13}C-^{1}H] HSQC, with only a small number of CβH_2 signals from aromatic residues (which were unaffected by ^{113}Cd substitution) in the same area of the spectrum. Comparison of the [^{13}C-^{1}H] HSQC spectrum of the (^{13}C, ^{15}N)-labelled Zn-bound protein with the natural abundance [^{13}C-^{1}H] HSQC spectrum of the ^{113}Cd-bound protein yielded unambiguous assignments for the CβH_2 signals of Cys 108, 122, 145, 148 and 153. For the remaining cysteine residues in the ^{113}Cd-bound protein (Cys 104, 105, 120 and 150), additional information came from analyzing the 2D [^{1}H-^{1}H] TOCSY and NOESY data, using the patterns of Hα-Hβ cross-peaks and comparing these to corresponding patterns for the Zn-bound protein; this analysis also yielded the Hα assignments for Cys 108, 122, 145, 148 and 153. Assignments made at 25°C were transferred also to 35°C by comparing natural abundance [^{13}C-^{1}H] HSQC data acquired at the two temperatures; shift differences between the two spectra were all small.

The metal-binding topology within $^{113}Cd_3$ Bud31p was determined as follows. The phase-sensitive [^{113}Cd, ^{113}Cd] COSY spectrum clearly shows that each of the three ^{113}Cd signals is coupled to both of the other two (even though cross-peaks to Cd2 are systematically weaker than others due to its broader linewidth, which in turn causes greater intensity loss due to anti-phase cancellation in the F_1 dimension). This establishes

unambiguously that the cadmiums form a cyclic three-metal cluster, which in turn implies that three of the nine metal-binding cysteines must each bridge between two cadmiums. To make sequence-specific assignments of cadmium-cysteine linkages, we used several [^{113}Cd, ^{1}H] experiments. The key question initially is to identify the three bridging cysteines. The HMQC-RELAY (at 35°C) shows clear cross-peaks linking both the Hβ2 and Hβ3 protons of Cys122 to Cd2 and Cd3 (main paper, Figure 1b); since both of these ^{1}H signals do not overlap with any other Cys signals, this establishes unambiguously that Cys122 is a bridging cysteine. The same spectrum also shows clear cross-peaks linking both Hβ2 and Hβ3 of Cys108 to both Cd1 and Cd2. The Hβ2 signal of Cys108 has a near-identical shift to that of Hβ3 of Cys145, and the Hβ3 signal of Cys108 has a near-identical shift to that of Hβ3 of Cys148. However, for both Cys145 and Cys148, it is clear that in each case the *other* Hβ signal shows no connectivity to one of the cadmiums (i.e. Cys145 Hβ2 does not link to Cd1, and Cys148 Hβ2 does not link to Cd2), and furthermore, Cys108 Hα shows cross peaks to both Cd1 (in HMQC-RELAY) and Cd2 (in HMQC-NOESY); this therefore establishes that it is Cys108 that bridges between Cd1 and Cd2. Clearly, the remaining bridging cysteine must link Cd1 and Cd3, and the only cysteinyl Hβ signal that appears for both Cd1 and Cd3 in the HMQC-RELAY spectrum is the Hβ2 signal of Cys104. Clear corroboration for the assignment of Cys104 as the Cd1-Cd3 bridge is seen in the HMQC-NOESY spectrum, where there are cross-peaks from Cys104 Hα (as well as weak peaks to Cys104 Hβ3) to both Cd1 and Cd3.

With the three bridging cysteines identified, assignment of the remaining two (non-bridging) cysteine ligands for each cadmium was straightforward. The HMQC-RELAY spectrum shows clear connections from Cd1 to Cys148 Hα and Cys105 Hβ2/3, and from Cd2 to Cys145 Hα and (more weakly) Cys120 Hβ2 and Hβ3, while Cd3 shows strong connections to Cys153 Hβ2 and Hβ3 and weak connections to Cys150 Hβ2/3 and Hα; these assignments are also corroborated by connections seen in the HMQC-NOESY spectrum. Furthermore, structures calculated from NOEs and J-couplings alone, with no metal

connectivites or ligand-ligand restraints in the input, produce structures that, although of low-resolution, have ligand-ligand distances that are all fully compatible with the assignments listed above.

SI.1.6 Structure Calculations.

NOE distance restraints were derived from analysis of all of the data from NOE-based experiments. Cross peaks intensities were measured using the program SPARKY[7] and grouped into four categories. Strong d_{NN} (i, i+1) connectivities in α-helices defined the category "strong" (0-2.8 Å, 361 constraints), $d_{\alpha N}$ (i, i+3) cross-peaks in helices defined the category "medium" (0-3.5 Å, 325 constraints), and all remaining peaks were classified as "weak" (0-5 Å, 1116 constraints), except for a small number of the very strongest peaks that were placed in the category "very strong" (0-2.3 Å, 26 constraints). Lower bounds for all NOE restraints were set to zero,[8] and no multiplicity corrections were required since r^{-6} summation was used for restraints involving groups of equivalent or non-stereoassigned spins.[9] Stereospecific assignments for 57 $C\beta H_2$ groups were made by analyzing HNHB, HACAHB and a short mixing time (τ_m = 50ms) ^{15}N-HSQC-NOESY spectra. Amide protons in stable hydrogen bonds were identified in a ^{15}N-HSQC spectrum recorded quickly after transferring a sample into ^2H_2O solution, and in cases where the corresponding acceptor was unambiguously identifiable in preliminary structures, distance restraints (0.0 Å < $d_{N...O}$ < 3.2 Å and 0.0 Å < $d_{H...O}$ < 2.2 Å) were applied during the structure calculations.

Structures were calculated from polypeptide chains with randomized ϕ and ψ torsion angles using a two-stage simulated annealing protocol within the program XPLOR-NIH.[10] In the first stage, no zinc ions were included in the calculations, and the arrangement of the zinc-binding ligands was imposed only by using inter-atomic ligand–ligand distances (Cys Sγ–

Cys Sγ, 3.7-3.9Å), specified according to the topology determined by the ^{113}Cd NMR results (Figures 2a and S5). These calculations employed initial Powell energy minimization (500 steps), dynamics at 1000K (20000 steps), increase of the van der Waals force constant and tilting of the NOE potential function asymptote (4000 steps), switching to a square-well NOE function then cooling to 300K in 2000 step cycles, and final Powell minimization (1000 steps). In the second stage, zinc ions were placed at the average of the four metal-binding atom positions in each case, linked according to the topology determined by the ^{113}Cd NMR results (Figures 2a and S5), and covalent constraints applied to impose correct local geometry during subsequent refinement. The structure was then subjected to initial Powell minimization (500 steps), increasing dihedral force constant during 4000 step cycles of dynamics at 1000K (with a strong van der Waals force constant and square-well NOE potential function), cooling to 300K in 1000 step cycles, and 2000 steps of final Powell minimisation. In all structure calculations, the force-field comprised only geometric terms; electrostatic, van der Waals attractive and generalized hydrogen bonding terms were not included.

The program CLUSTERPOSE[11] was used to calculate the mean rmsd of ensembles to their mean structure, and structures were visualised using the program PYMOL.[12]

**Structural restraints**				
NOE-derived distance restraints				
Intraresidue	676	V. Strong	(0 - 2.3Å)	26
Sequential	408	Strong	(0 - 2.8Å)	361
Medium (2≤|i-j|≤4)	390	Medium	(0 - 3.5Å)	325
Long (|i-j|>4)	354	Weak	(0 - 5.0Å)	1116
Total	1828			
Dihedral restraints				
phi		70		
psi		70		
Chi1		57		
H-bonds restraints		30 distances<br>15 H-bonds		
**Statistics for accepted structures**				
Number of accepted structures		35		
Mean XPLOR energy terms (kcal mol^{-1} ± S.D.)				
E(total)		1007.1 ± 12.4		
E(van der Waals)		422.0 ± 3.8		
E(distance restraints)		42.7 ± 3.6		
E(dihedral restraints)		0.46 ± 0.14		
Distance restraint viols. > 0.2Å (average number per structure)		1.7 ± 0.9		
Angle restraint viols. > 5° (average number per structure)		11.4 ± 1.6		
**RMS deviations from the ideal geometry used within XPLOR**				
Bond lengths		0.0066 Å		
Bond angles		0.89°		
Improper angles		0.37°		
**Ramachandran Statistics**				
Most favoured		83.2%		
Additionally allowed		15.6%		
Generously allowed		0.9%		
Disallowed		0.3%		
**Average atomic RMS deviations from the average structure (± S.D.)**		Res. 12-36, 45-109, 119-155		
(N, C^{α}, C atoms)		0.52 ± 0.17 Å		
(All heavy atoms)		0.91 ± 0.13 Å		

Table S1 Structural statistics for the deposited ensemble of BUD31p structures.

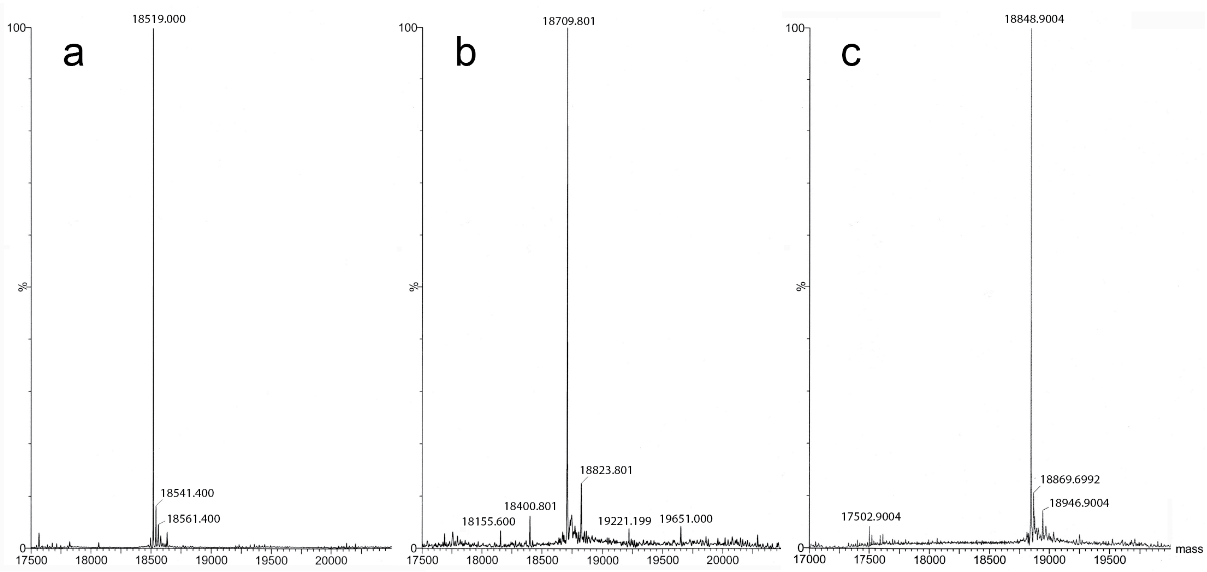

Figure S1

Mass spectra of Bud31p under denaturing and non-denaturing conditions to verify the metal content. a) Bud31p under denaturing conditions without metal bound (principal peak 18519.000); b) Zn_3 Bud31p measured under non-denaturing conditions (principal peak 18709.801); c) $^{113}Cd_3$ Bud31p measured under non-denaturing conditions (principal peak 18848.9004). The mass difference between the zinc and the cadmium species of Bud31p indicate a full exchange of zinc for cadmium.

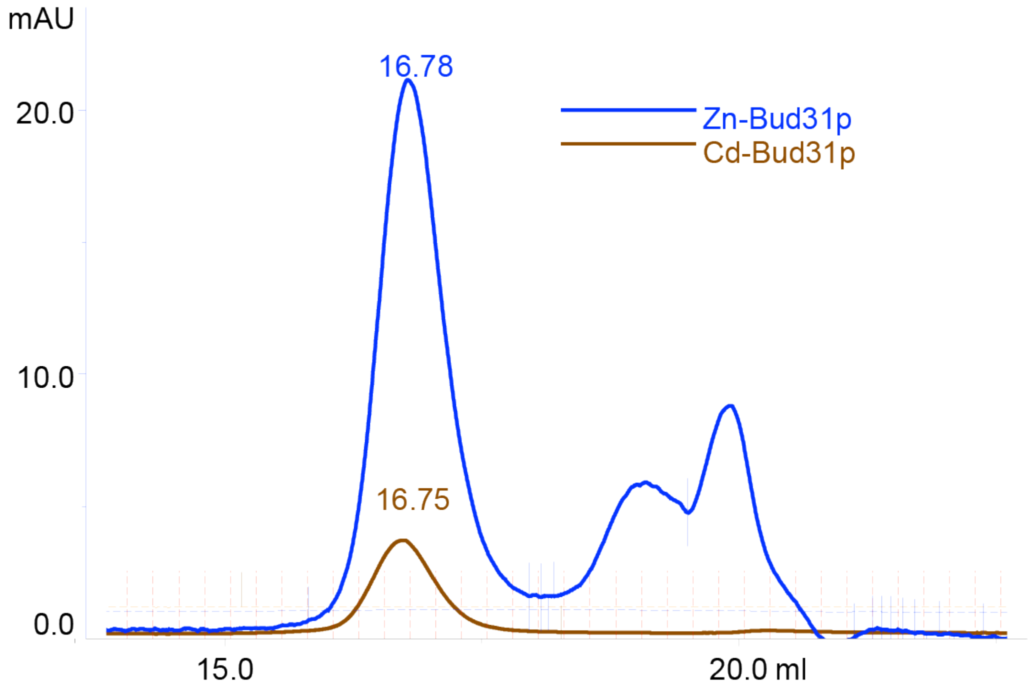

Figure S2

Gel filtration results for Bud31. Overlay of gelfiltration runs; Zn_3 Bud31p (blue) and $^{113}Cd_3$ Bud31p (brown) virtually overlap at an elution volume around 16.8 ml, indicating that substitution of zinc for cadmium did not alter the overall size and shape of the protein. No protein aggregates were observed in the void volume of the column (Superdex S200 HR 10/300 column). The additional peaks in the blue trace at 19-20 ml stem from a slightly different buffer system.

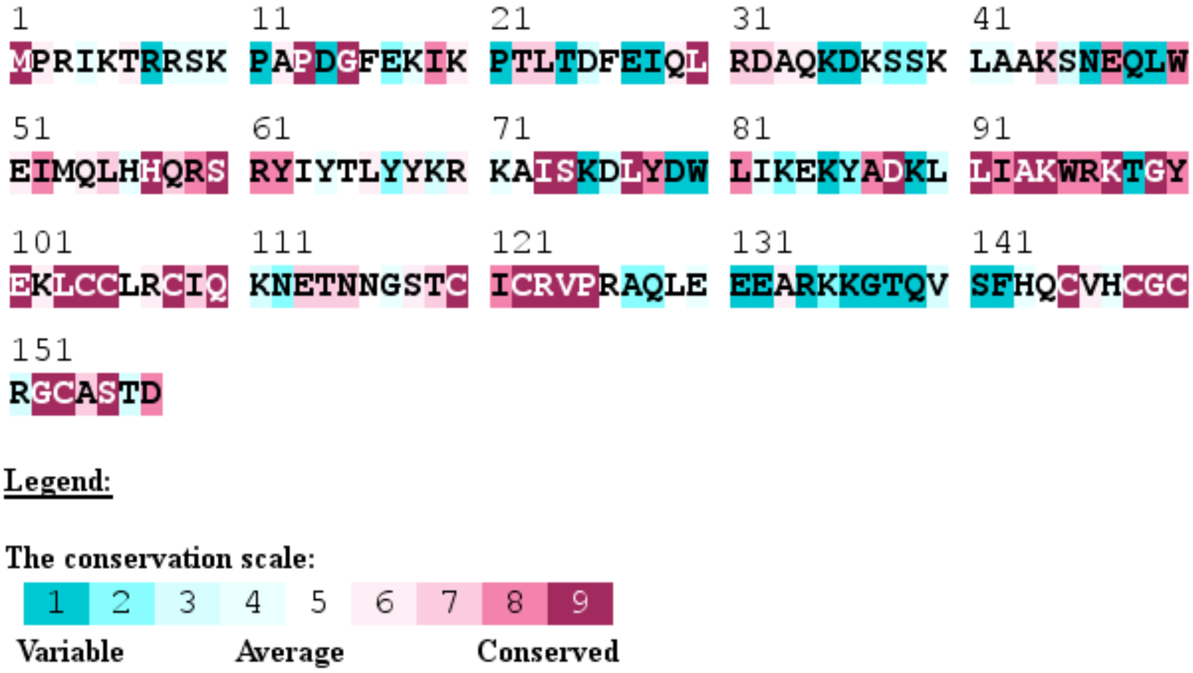

Figure S3

The sequence of Bud31p, colored according to CONSURF conservation scores. The conservation scores were calculated with CONSURF,[13] using a manually curated multiple sequence alignment of 316 Bud31p homologs. The homologous sequences were collected from a PSI-BLAST [14] search against the NCBI NR sequence database, then aligned with MUSCLE [15] and manually edited using JALVIEW.[16] All nine of the metal-binding cysteine residues are invariant across species.

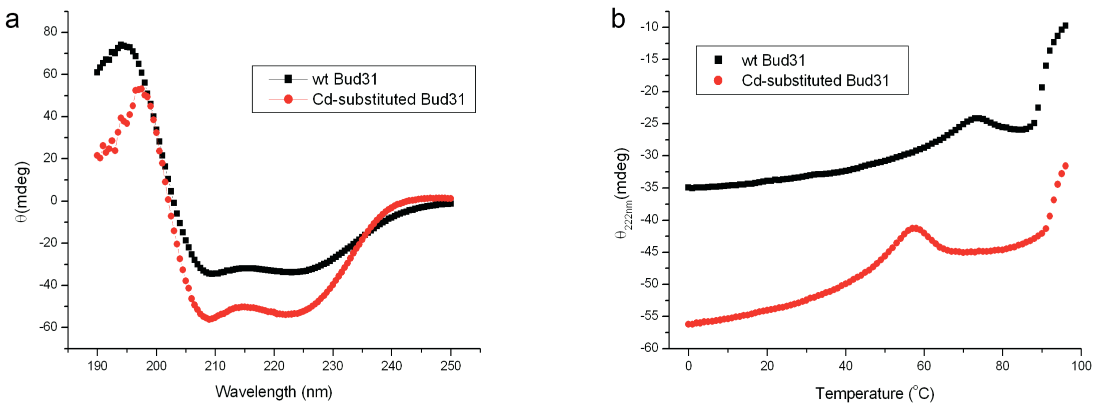

Figure S4

CD and thermal denaturation of Bud31p. a) Overlay of the UV-CD spectra of Zn_3 Bud31p (black) and $^{113}Cd_3$ Bud31p (red), 20°C. The spectra overlay very well indicating the secondary structure is largely unchanged upon substitution of zinc for cadmium. b) Overlay of thermal denaturations of Zn_3 Bud31p (black) and $^{113}Cd_3$ Bud31p (red) show that the cadmium substituted protein starts to denature at a much lower temperature, indicating that it is less stable.

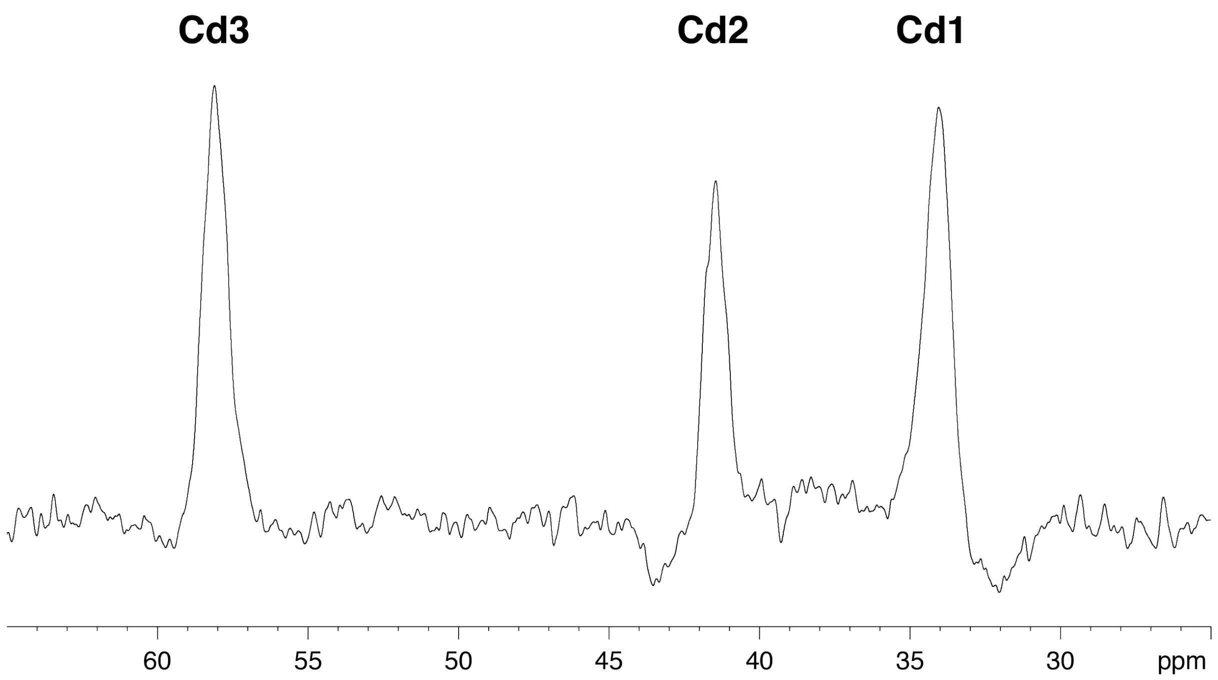

Figure S5

One-dimensional ^{113}Cd NMR spectrum of $^{113}Cd_3$ Bud31p (2.6 mM in $^{2}H_2O$), recorded at 25°C on a Bruker AVIII HD 500 MHz spectrometer (111.0 MHz for ^{113}Cd). This spectrum results from 2048 scans and took 47 min. to acquire.

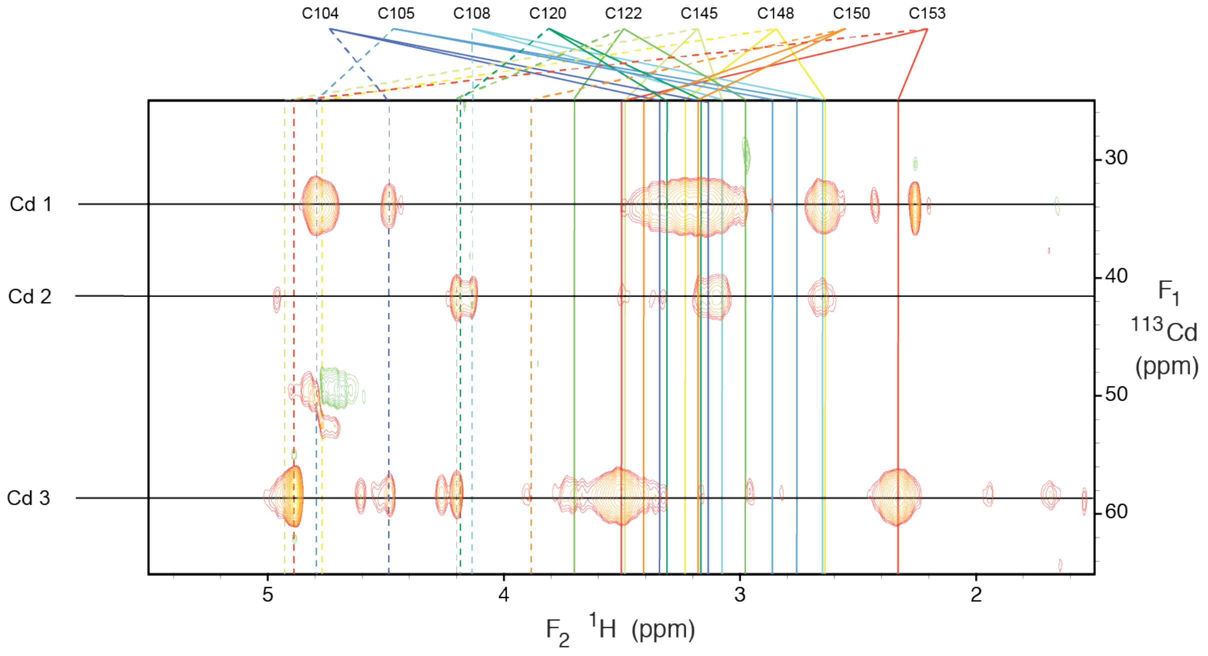

Figure S6

Two-dimensional [^{113}Cd, ^{1}H] HMQC-NOESY spectrum of $^{113}Cd_3$ Bud31p (2.6 mM in $^{2}H_2O$), recorded at 25°C on a Bruker AVIII HD 500 MHz spectrometer (111.0 MHz for ^{113}Cd). Assignments of Cys Hβ (solid lines) and Hα (dotted lines) signals are shown. This experiment employed 32 t_1-increments, each with 2048 scans, and took 21 hrs. to acquire.

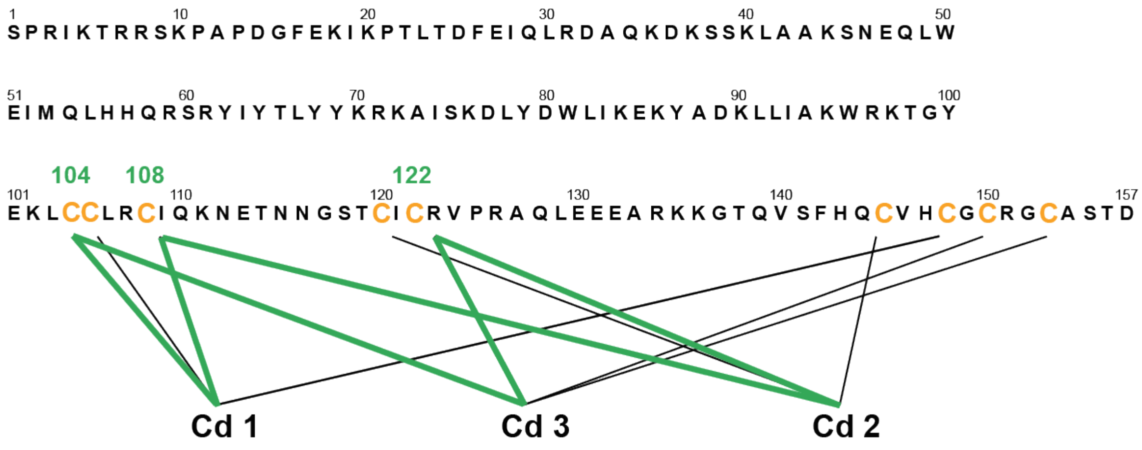

Figure S7

Metal binding topology of Bud31p as determined by ^{113}Cd NMR experiments. This view shows the same information as Figure 2a of the main paper, re-arranged to emphasize sequential relationships amongst the cysteine ligands. Bonds to bridging cysteines are shown in green.

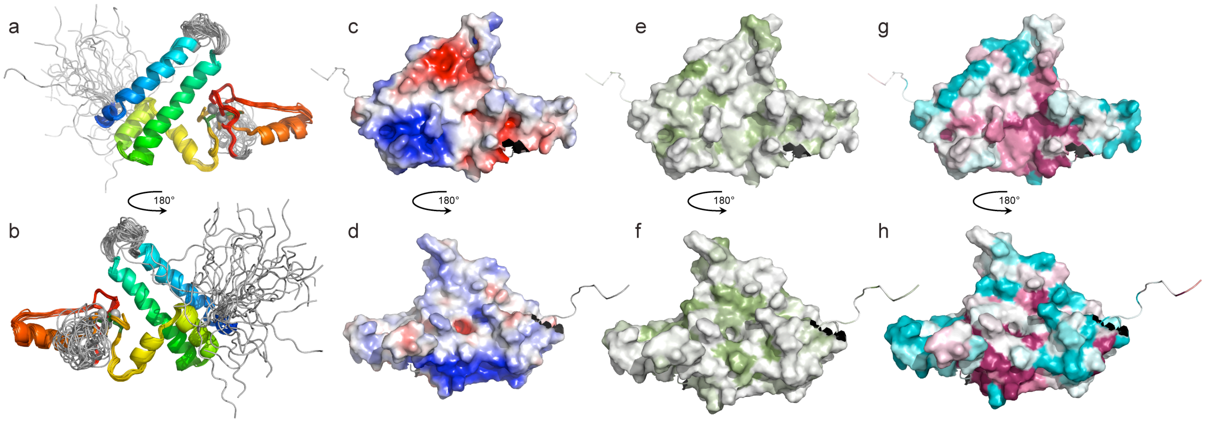

Figure S8

Overall views of the structure of Bud31p. a), b) Ensemble views, generated using the program Clusterpose.[11b] Chainbow colouring is used, running from blue at the N-terminus to red at the C-terminus (disordered regions as defined in Figure S7 are shown in grey). c)-h) Surface views shown using the lowest NOE energy structure: c), d) Potential surface calculated using the program and APBS,[17] coloring runs from red (-4.0 K_bT/e_c) to blue (+4.0 $K_bT/e_{c,}$); e), f) Hydrophobic surface colored according to the Kyte-Doolittle scale;[18] g), h) Conservation scores mapped to the protein surface (cyan, variable; maroon, conserved); conservation scores and coloring scale are as described in the caption to Figure S3.

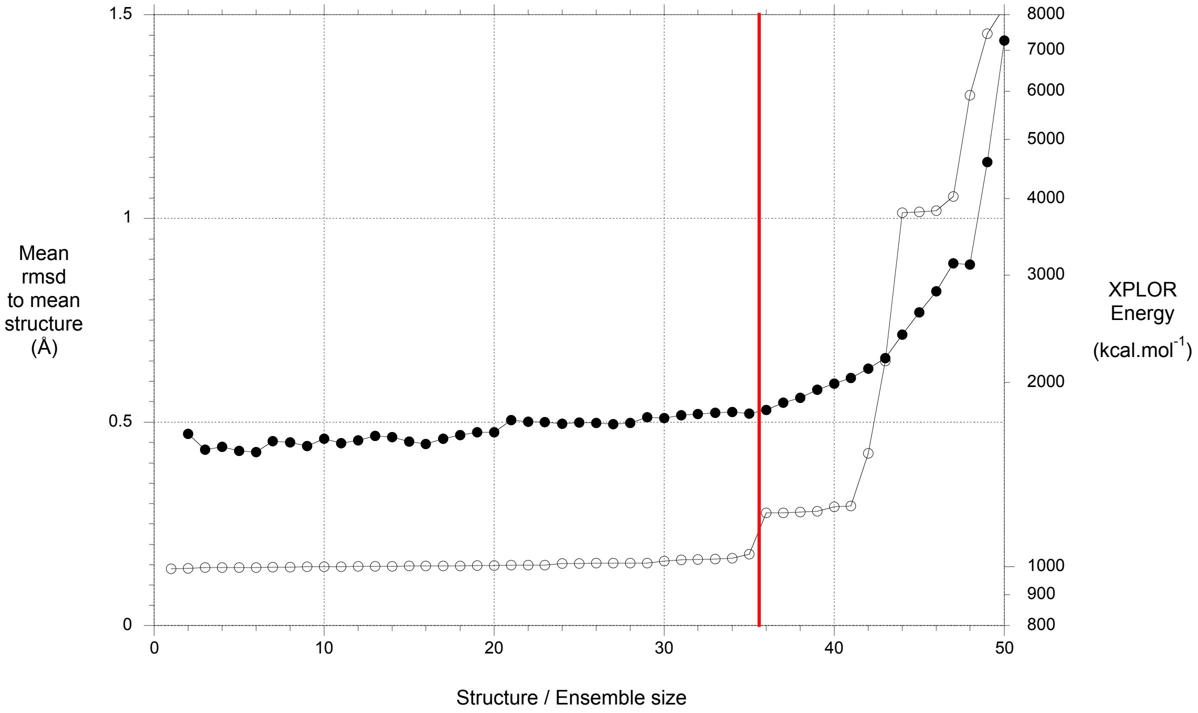

Figure S9

Rmsd and XPLOR energy profiles for the 50 calculated structures of Bud31p. Rmsd values (filled circles) are independently calculated for each ensemble size using the program CLUSTERPOSE,[11] adding successive structures in order of increasing XPLOR energy; open circles represent the XPLOR energies of each structure. Only the 35 structures to the left of the vertical red line were included in the deposited ensemble and when calculating the structural statistics.

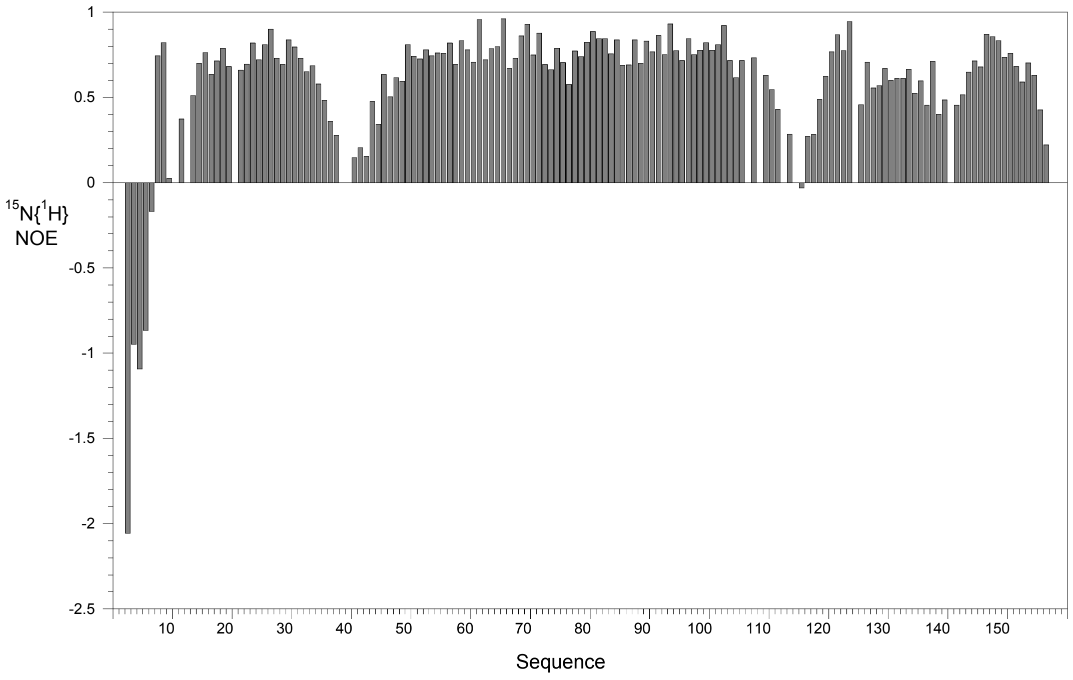

Figure S10

Steady-state $^{15}N\{^{1}H\}$ NOE data for Bud31p, obtained at 600 MHz and 27°C using the pulse sequence of Farrow *et al.*[19] In addition to the N-terminal tail, there are clear regions of reduced NOE values corresponding to the flexible loops at approximately residues 37-44 and 108-120, as well as slightly reduced values for residues 125-135.

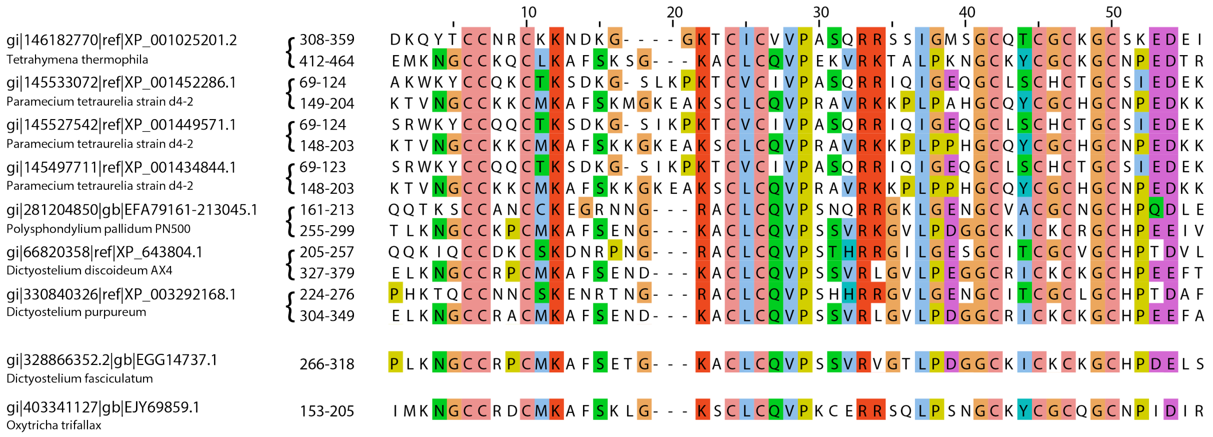

Figure S11

Sequence alignment of metal-binding domain homologous to that in Bud31p found in some protozoa and amoebi. In this small family the metal-binding domain occurs without the associated helical bundle domain seen for Bud31p, and in all but the last two cases, two copies of the domain occur as a closely-spaced tandem repeat.

Supplementary References:

[1] D. S. Wishart, Bigam, C. G., Yao, J., Abildaard, F., Dyson, H. J., Oldfield, E., Markley, J. L., Sykes, B. D., *J. Biomol. NMR* **1995**, *6*, 135-140.

[2] R. K. Harris, E. D. Becker, S. M. C. De Menezes, R. Goodfellow, P. Granger, *Pure Appl Chem* **2001**, *73*, 1795-1818.

[3] M. H. Frey, G. Wagner, M. Vasak, O. W. Sørensen, D. Neuhaus, E. Wörgötter, J. H. R. Kägi, R. R. Ernst, K. Wüthrich, *J. Am. Chem. Soc.* **1985**, *107*, 6847-6851.

[4] T. J. Norwood, J. Boyd, J. E. Heritage, N. Soffe, I. D. Campbell, *J. Magn. Reson.* **1990**, *87*, 488-501.

[5] J. D. Otvos, I. M. Armitage, *Proc. Natl. Acad. Sci. U. S. A.* **1980**, *77*, 7094-7098.

[6] a) O. Zerbe, D. L. Pountney, W. Vonphilipsborn, M. Vasak, *J. Am. Chem. Soc.* **1994**, *116*, 377-378; b) O. Zerbe, D. L. Pountney, W. Vonphilipsborn, M. Vasak, *J. Am. Chem. Soc.* **1994**, *116*, 7957-7957.

[7] T. D. Goddard, Kneller, D. G., University of California, San Francisco.

[8] U. Hommel, T. S. Harvey, P. C. Driscoll, I. D. Campbell, *Journal of Molecular Biology* **1992**, *227*, 271-282.

[9] a) M. Nilges, *Proteins* **1993**, *17*, 297-309; b) C. M. Fletcher, Jones, D. N. M., Diamond, R., Neuhaus, D., *J. Biomol. NMR* **1996**, *8*, 292-310.

[10] C. D. Schwieters, J. J. Kuszewski, N. Tjandra, G. M. Clore, *J. Magn. Reson.* **2003**, *160*, 65-73.

[11] a) R. Diamond, *Protein Sci.* **1992**, *1*, 1279-1287; b) R. Diamond, *Acta Cryst. D* **1995**, *51*, 127-135.

[12] W. L. DeLano, DeLano Scientific, Palo Alto, CA, **2002**.

[13] F. Glaser, T. Pupko, I. Paz, R. E. Bell, D. Bechor-Shental, E. Martz, N. Ben-Tal, *Bioinformatics* 2003, *19*, 163-164.

[14] S. F. Altschul, T. L. Madden, A. A. Schaffer, J. Zhang, Z. Zhang, W. Miller, D. J. Lipman, *Nucleic Acids Res.* 1997, *25*, 3389-3402.

[15] R. C. Edgar, *Nucleic Acids Res.* 2004, *32*, 1792-1797.

[16] A. M. Waterhouse, J. B. Procter, D. M. Martin, M. Clamp, G. J. Barton, *Bioinformatics* 2009, *25*, 1189-1191.

[17] N. A. Baker, D. Sept, S. Joseph, M. J. Holst, J. A. McCammon, *Proc. Natl. Acad. Sci. U. S. A.* 2001, *98*, 10037-10041.

[18] J. Kyte, R. F. Doolittle, *J. Mol. Biol.* 1982, *157*, 105-132.

[19] N. A. Farrow, R. Muhandiram, A. U. Singer, S. M. Pascal, C. M. Kay, G. Gish, S. E. Shoelson, T. Pawson, J. D. Forman-Kay, L. E. Kay, *Biochemistry* 1994, *33*, 5984-6003.
